# Prevalence of complications in eyes with nanophthalmos or microphthalmos: protocol for a systematic review and meta-analysis

**DOI:** 10.1186/s13643-022-01889-5

**Published:** 2022-02-09

**Authors:** Naseer Ally, Sarah Ismail, Hassan Dawood Alli

**Affiliations:** Department of Neurosciences, Division of Ophthalmology, University of the Witwatersrand, 7 York Road, Parktown, Johannesburg, Gauteng 2193 South Africa

**Keywords:** Microphthalmos, Nanophthalmos, Complications, Hyperopia, Uveal effusion, Glaucoma

## Abstract

**Introduction:**

Microphthalmos and nanophthalmos are uncommon ocular conditions, whereby affected eyes have smaller dimensions compared to the normal population. Microphthalmos and nanophthalmos present several challenges to ophthalmologists; they have spontaneous and post-operative sequelae such as high hyperopia, angle-closure glaucoma, uveal effusion syndrome, and retinal detachment.

This systematic review and meta-analysis intends to assess the prevalence of both the spontaneous complications associated with nanophthalmos and microphthalmos, as well as the post-surgical complications associated with nanophthalmos or microphthalmos.

**Methods and analysis:**

Articles will be searched for, on four online databases: PubMed, EMBASE, Scopus, and Web of Science. Two independent reviewers will identify the studies according to prespecified inclusion and exclusion criteria. All studies included with participants diagnosed with microphthalmos or nanophthalmos in one or both eyes, will be included if they have (i) more than 4 cases and (ii) defined microphthalmos/nanophthalmos as an axial length of < 21 mm or a high lens/eye volume ratio. Nanophthalmos may have an additional diagnostic criterion of posterior wall thickness greater than 1.7 mm. The prevalence of the following complications will be assessed: high hyperopia (spherical equivalent >3D), angle closure glaucoma, uveal effusion syndrome, retinal detachment, and chorioretinal folds.

Studies that will be excluded are those that have not adequately defined the criteria for the diagnosis of nanophthalmos or microphthalmos, those studies that have less than five cases, studies with criteria not defined above, and deemed unsuitable, and studies in languages other than English with no published translation.

Relevant data will be extracted and assessed for the risk of bias in each article using a modified Joanna Briggs Institute (JBI) assessment tool. The data will then be pooled to determine the prevalence of complications among patients with microphthalmos and nanophthalmos. If the data allows, subgroup analysis will be carried out according to axial length as well as subtype of microphthalmos/nanophthalmos (simple, complex, relative anterior, and posterior).

**Discussion:**

Although nanophthalmos is an uncommon condition that affects the eye, its management and complications can be sight-threatening. Thus, it is important to counsel patients and their families correctly (in the case of children) upon diagnosis and prior to any surgical intervention. This can only be done if the overall prevalence of complications is known.

**Registration:**

PROSPERO CRD42021227847

## Background

Microphthalmos and nanophthalmos are uncommon ocular conditions where the size of the eye is smaller than that of the normal population [1–3]. Axial length, the parameter most commonly used to measure the ocular size, is shorter in these eyes. Microphthalmos is divided into simple microphthalmos which has no associated ocular malformations, or complex microphthalmos, in which ocular malformations or systemic syndromes are present [1, 3]. Nanophthalmos is a subset of simple microphthalmos where, in addition to the shorter axial length, the eye also has a thickened sclera and choroid [2]. This is thought to occur as a result of an abnormality in the arrangement of the collagen fibrils [4]. The terms microphthalmos and nanophthalmos are often used interchangeably and the absolute limit for the definition of shortened axial length varies and is the subject of debate [5]. It is usually taken as an axial length of less than two standard deviations from the normal for age [6]. The absolute limit described in the literature are < 21.0 mm [5, 7], < 20.9 mm [6], < 20.5 mm [8, 9], < 20mm [10], < 18.5 mm [3], and < 17 mm [11]. When there is a relative shortening of either the anterior or posterior segments of the eye, this is known as relative anterior or posterior microphthalmos respectively [1]. Other parameters measured for microphthalmos and/or nanophthalmos include; shallow anterior chamber, high hyperopia, posterior wall thickness greater than 1.7 mm, and a high lens/eye volume ratio [1, 2].

Microphthalmos and nanophthalmos present several vision-related challenges to ophthalmologists which can occur spontaneously or after surgery. Spontaneous vision-related problems are high hyperopia, angle-closure glaucoma, uveal effusion syndrome, and retinal detachment [1, 2]. Other associations include corneal steepening, enlarged foveal avascular zone, optic disc drusen, central retinal vein occlusion, and chorioretinal folds [1, 2, 11].

Surgery in nanophthalmos and microphthalmos is associated with higher rates of complications and poorer visual outcomes [12]. Cataract surgery is associated with complications such as anterior uveitis, uveal effusions, corneal decompensation, retinal detachment, cystoid macular oedema, choroidal haemorrhage, vitreous haemorrhage, and aqueous misdirection [11, 13]. Management strategies to prevent these post-operative complications include the use of pre-operative steroids, pre-operative mannitol infusions, peripheral iridotomies, and scleral lamellar resections [9]. Glaucoma surgery is also fraught with potential complications which include uveal effusion, choroidal folds, and cataract formation.

To date, reviews have been conducted which looked at the clinical spectrum and the treatment of complications in nanophthalmos. However, to our knowledge, there has been no systematic review looking at the prevalence of these complications among patients with microphthalmos or nanophthalmos. These clinical entities are uncommon, and therefore, it is often difficult to quantify the prevalence of complications in these conditions. This is of importance clinically for two reasons. Firstly, when diagnosing a patient with the condition they need to be counselled on the natural history and potential complications associated with the condition. Secondly, when patients with this condition require surgery, they need to be counselled pre-operatively so that they know what the percentages of complications post-operatively are. We, therefore, intend to undertake a systematic review and meta-analysis assessing the prevalence of both the spontaneous and post-surgical complications associated with nanophthalmos and microphthalmos. We hope that this will be the first step in answering the question regarding the prevalence of complications in this condition.

## Methods

### Study design

This systematic review will include retrospective and prospective case series, cross-sectional studies, retrospective and prospective cohorts, and randomized clinical trials. It will be conducted according to the PRISMA-P (Preferred Reporting Items for Systematic Review and Meta-Analysis Protocol) guidelines [14]. This systematic review has also been registered on PROSPERO [15].

### Inclusion criteria

The proposed inclusion criteria for this systematic review are as follows:

- Population: All studies, with participants diagnosed with microphthalmos or nanophthalmos in one or both eyes, will be included if they have (i) more than 4 cases and (ii) defined microphthalmos/nanophthalmos as an axial length of < 21 mm or a high lens/eye volume ratio. Nanophthalmos may have an additional diagnostic criterion of posterior wall thickness greater than 1.7 mm.
- Condition: The prevalence of the following complications will be assessed: high hyperopia (spherical equivalent >3D) [16], angle closure glaucoma, uveal effusion syndrome, retinal detachment, and chorioretinal folds [1, 2]. Spontaneous ocular complications are defined as those ocular complications that are diagnosed prior to any surgical procedure performed. If a surgical procedure occurred prior to the diagnosis, the study would fall under post-surgical complications.
- Context: The studies will probably be hospital-based cohorts or case series. If population-based studies are found these will be included and analysed separately.

Since this is a prevalence study, no comparison group is necessary.

### Exclusion criteria

Studies that will be excluded are (i) those that have not adequately defined the criteria for the diagnosis of nanophthalmos or microphthalmos, (ii) studies that have less than five cases, (iii) studies with criteria not defined above, and deemed unsuitable, and (iv) studies in languages other than English with no published translation.

### Databases and information sources

Four databases will be searched for relevant studies: PubMed, EMBASE, Web of Science, and Scopus. Registered clinical trials that are published will also be sought on ClinicalTrials.gov. Reviews will be checked on the Cochrane database. The reference lists of these reviews and any reviews found on the other databases will be checked for additional articles. Where necessary, authors will be contacted for clarification by one reviewer (NA).

### Search strategy

The search strategy, using the search terms in Appendix A, has been developed using the CoCoPop method [17]. All the information and data will be collated and entered onto Microsoft Excel (Microsoft Corporation). The data collected will be assessed on the first 5 articles and amended if required.

### Study selection

**Fig. 1:**
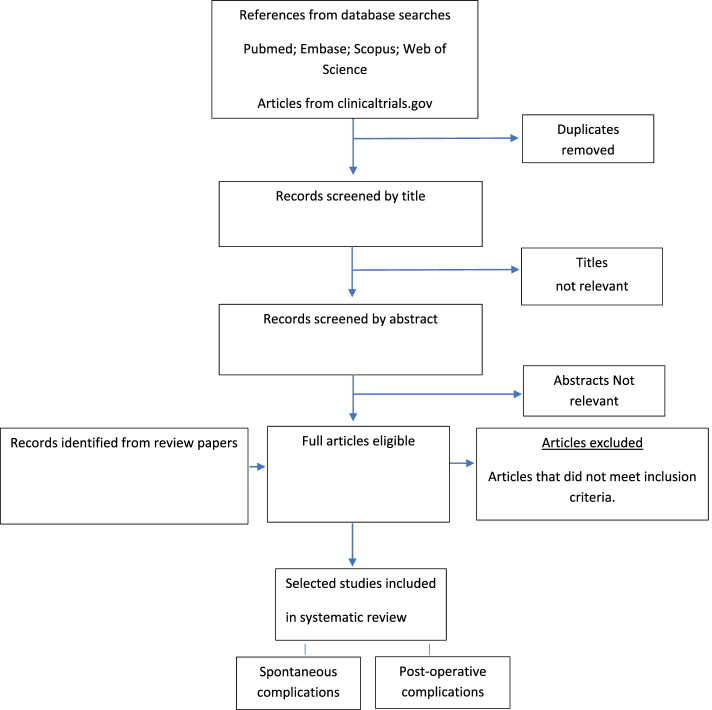
PRISMA flow diagram

The study selection process will be conducted using the PRISMA guidelines and flow diagram [14]. After exclusion of duplicate studies using the Zotero™ citation manager, and after being rechecked manually, all titles and abstracts will be reviewed independently by two reviewers (NA and SI) according to the prespecified inclusion and exclusion criteria (Fig. 1 in Appendix B). Any disagreements will be resolved by a third reviewer (HDA). Systematic and narrative reviews, animal studies, editorials and letters will be excluded. The reference lists of review papers will be screened for studies that meet the inclusion criteria. The full texts of eligible studies/papers will be examined for inclusion into the systematic review and meta-analysis (Fig. 1 in Appendix B). When data from the same cohort are reported in separate manuscripts, the study reporting the largest sample fulfilling our eligibility criteria will be selected. If there are doubts regarding these datasets, the corresponding authors will be contacted for clarification.

Studies that are included, and decisions made, will be recorded on Microsoft Excel™ (Microsoft Corporation).

### Risk of bias assessment/quality assessment

Studies assessing the prevalence of spontaneous complications and post-operative complications in cohorts and case series will be assessed using a modified version of the Joanna Briggs Institute (JBI) critical appraisal tool [17, 18].

### Data extraction

Data will be extracted by one reviewer (NA), checked by another reviewer (SI), and populated onto Microsoft Excel™ (Microsoft Corporation). Disagreements will be resolved by a third reviewer (HDA). The following data will be extracted: year of study, study design (i.e. cohort, cross-sectional etc.), total number of participants, axial length, proportion of males to females, mean/median age at presentation, number of participants with one or more complications (high hyperopia [spherical equivalent >3D], presence of cataract, angle closure glaucoma, uveal effusion syndrome, retinal detachment, and chorioretinal folds), posterior wall thickness, and lens/eye volume ratio.

### Outcomes

The outcomes that will be calculated and analysed will be the proportion of patients with cataract, angle closure glaucoma, uveal effusion syndrome, retinal detachment, and chorioretinal folds.

### Data synthesis

Data will be meta-analysed if there are at least two studies that report a specific complication, to allow the pooling of participants with either nanophthalmos or microphthalmos.

### Data analysis

Data will be analysed in Stata 16.1 (STATACorp LLC, College Station, Texas). Due to the difference in definitions and diagnosis of microphthalmos and nanophthalmos, random effects models will be used throughout. The presence of spontaneous complications will be analysed using the “metaprop” command in Stata. The Freeman-Tukey double arcsine transformation will be performed to normalize outcomes before pooling the prevalence. Study specific 95% confidence intervals will be generated using the exact method. The *I*^*2*^ statistic will be used to check for overall, intergroup, and intragroup heterogeneity. Forest plots will then be generated from the data.

For post-operative complications where a comparison group is used, the effect size will be compared using the “meta set” command. A random effects model will be used to present the overall effect size as an odds ratio with 95% confidence intervals. Forest plots will then be generated from the data.

### Subgroups

Subgroups will be analysed according to axial length to see if the proportion of complications increases/decreases with decreasing axial length in both the spontaneous complication group and post-surgical complication group. Subgroups will also be analysed according to subtypes of microphthalmos (anterior, posterior, simple and complex), if the data permits this.

### Meta-bias

Due to the low prevalence of these conditions, we anticipate most of the studies will be cohort or case-series studies. Assessing publication bias will be challenging due to a paucity of RCT data. Selective reporting bias will be assessed using the JBI critical appraisal tool [17, 18].

### Strength of evidence

After the studies are assessed using the JBI critical appraisal tool, the data will be re-analysed and forest plots will be generated from studies that do not have a low quality of evidence.

### Data availability

Data will be made available upon request to the corresponding author.

## Discussion

Although microphthalmos and nanophthalmos are uncommon conditions that affect the eye, its management and complications can be sight-threatening [1, 2, 8, 12]. Due to the conditions being uncommon, it is difficult to perform single-centre studies with large patient numbers. A systematic review and meta-analysis provides a valid method to assess the prevalence of complications in these patients. It is also important to counsel these patients correctly upon diagnosis and prior to any surgical intervention. Thus far, to our knowledge, there is no systematic review and meta-analysis assessing the prevalence of complications of patients with microphthalmos or nanophthalmos, that occur spontaneously, or after surgery. We believe that this will add valuable information to the body of knowledge on the subject.

### Strengths and weaknesses

This protocol is in line with the PRISMA guidelines for the conducting of systematic reviews and it has been registered on PROSPERO. Amendments to his protocol will be updated on the PROSPERO register.

Weaknesses include the inclusion of only English articles which means that articles in other languages may be missed, and possible heterogeneity in methodology (especially the definitions of microphthalmos/nanophthalmos) and data.

## Data Availability

Not applicable

## Appendix A

### Search strategy

“microphthalmos”[MeSH Terms] OR “microphthalmos”[All Fields] OR (“microphthalmos”[MeSH Terms] OR “microphthalmos”[All Fields] OR “nanophthalmos”[All Fields]) OR ((“microphthalmos”[MeSH Terms] OR “microphthalmos”[All Fields] OR (“microphthalmos”[MeSH Terms] OR “microphthalmos”[All Fields] OR “nanophthalmos”[All Fields])) AND (“complications”[All Fields] OR “complicate”[All Fields] OR “complicated”[All Fields] OR “complicates”[All Fields] OR “complicating”[All Fields] OR “complication”[All Fields] OR “complication s”[All Fields] OR “complications”[MeSH Subheading] OR “complications”[All Fields])) OR ((“microphthalmos”[MeSH Terms] OR “microphthalmos”[All Fields] OR (“microphthalmos”[MeSH Terms] OR “microphthalmos”[All Fields] OR “nanophthalmos”[All Fields])) AND (“surgery”[MeSH Subheading] OR “surgery”[All Fields] OR “surgical procedures, operative”[MeSH Terms] OR (“surgical”[All Fields] AND “procedures”[All Fields] AND “operative”[All Fields]) OR “operative surgical procedures”[All Fields] OR “general surgery”[MeSH Terms] OR (“general”[All Fields] AND “surgery”[All Fields]) OR “general surgery”[All Fields] OR “surgery s”[All Fields] OR “surgeries”[All Fields] OR “surgeries”[All Fields])) OR ((“microphthalmos”[MeSH Terms] OR “microphthalmos”[All Fields] OR (“microphthalmos”[MeSH Terms] OR “microphthalmos”[All Fields] OR “nanophthalmos”[All Fields])) AND (“epidemiology”[MeSH Subheading] OR “epidemiology”[All Fields] OR “prevalence”[All Fields] OR “prevalence”[MeSH Terms] OR “prevalence”[All Fields] OR “prevalences”[All Fields] OR “prevalence s”[All Fields] OR “prevalent”[All Fields] OR “prevalently”[All Fields] OR “prevalents”[All Fields])) OR ((“microphthalmos”[MeSH Terms] OR “microphthalmos”[All Fields] OR (“microphthalmos”[MeSH Terms] OR “microphthalmos”[All Fields] OR “nanophthalmos”[All Fields])) AND (“glaucoma”[MeSH Terms] OR “glaucoma”[All Fields] OR “glaucomas”[All Fields])) OR ((“microphthalmos”[MeSH Terms] OR “microphthalmos”[All Fields] OR (“microphthalmos”[MeSH Terms] OR “microphthalmos”[All Fields] OR “nanophthalmos”[All Fields])) AND ((“uvea”[MeSH Terms] OR “uvea”[All Fields] OR “uveal”[All Fields]) AND (“effusate”[All Fields] OR “effusates”[All Fields] OR “effused”[All Fields] OR “effusion”[All Fields] OR “effusions”[All Fields] OR “effusive”[All Fields]))) OR ((“microphthalmos”[MeSH Terms] OR “microphthalmos”[All Fields] OR (“microphthalmos”[MeSH Terms] OR “microphthalmos”[All Fields] OR “nanophthalmos”[All Fields])) AND (“hyperopia”[MeSH Terms] OR “hyperopia”[All Fields] OR “hyperopias”[All Fields])) OR ((“microphthalmos”[MeSH Terms] OR “microphthalmos”[All Fields] OR (“microphthalmos”[MeSH Terms] OR “microphthalmos”[All Fields] OR “nanophthalmos”[All Fields])) AND (“therapeutics”[MeSH Terms] OR “therapeutics”[All Fields] OR “treatments”[All Fields] OR “therapy”[MeSH Subheading] OR “therapy”[All Fields] OR “treatment”[All Fields] OR “treatment s”[All Fields])) OR ((“microphthalmos”[MeSH Terms] OR “microphthalmos”[All Fields] OR (“microphthalmos”[MeSH Terms] OR “microphthalmos”[All Fields] OR “nanophthalmos”[All Fields])) AND (“therapeutical”[All Fields] OR “therapeutically”[All Fields] OR “therapeuticals”[All Fields] OR “therapeutics”[MeSH Terms] OR “therapeutics”[All Fields] OR “therapeutic”[All Fields])

## Abbreviations

PRISMA-P: Preferred Reporting Items for Systematic Review and Meta-Analysis Protocol
JBI: Joanna Briggs Institute
